# Extracellular vesicles in fetal-maternal immune tolerance

**DOI:** 10.1016/j.bj.2024.100785

**Published:** 2024-08-29

**Authors:** William J. Burlingham

**Affiliations:** University of Wisconsin, Wisconsin, USA

**Keywords:** Immunology, Tolerance, Exosomes, Pregnancy

## Abstract

Two key problems of allo-tolerance during fetal-maternal co-existence are: 1) it's focus must be local, allowing the mother's continued peripheral immune competence to resist pathogens ubiquitously, and 2) it must propagate itself, i.e. continuously recruit new re-enforcements of the local tolerant state. Both are solved by the exosomal pathway of Tregs & Bregs. While the fetal-maternal accomodations of pregnancy terminate at the time of partrurition, geography, climate and the endemic pathogens of the environment surrounding the mother-baby pair would then define the short and long-term effects of their immunologic interaction.

## 1 What is meant by the term: fetal-maternal immune tolerance?

Pregnancy in viviparous mammals presents the greatest challenge to the survival of such species. The carriage of a semi-allogeneic fetus within the body of the mother until the point of viability outside of the womb required several layers of immune development over millions of years of evolution. Tolerance to self but absolute immunity to non-self governs immune system development in fish, reptiles, and oviparous mammals. However, over the course of evolution, the viviparous mammal mother's immune system and anatomy needed to incorporate a major exception to this rule: how NOT to reject the semi-allogeneic fetus. For the term of pregnancy, a period of between 19 and 21 days in mice to nearly two years (22 months) in elephants, the fully developed maternal immune system and the emerging immune system of the fetus must have strategies to accept what would otherwise be seen as a recognizable threat. From the point of view of the mother, the fetus, which has inherited her antigens but also expresses the antigens inherited from the father (termed “IPA, or inherited paternal alloantigens”), looks “different”. From the point of view of the fetus, the antigens inherited from mother (termed “IMA, or inherited maternal alloantigens”) are “self” and therefore no problem. However, during pregnancy the baby's developing immune system is surrounded by, and to a limited extent even after birth, is infiltrated by maternal cells that express antigens that they did NOT inherit (so-called “NIMA, or non-inherited maternal antigens). Accomodations to these differences insure a safe pregnancy for mother and fetus, while avoiding pregnancy loss due to aberrant or premature immune reactions to MHC and minor H antigen mismatches.

The long-term effects of these temporary immune “blindspots“ of the mother and newborn, are quite different after parturition. Both mother and offspring become free to develop self/non-self discrimination as oviparous mammals do. However, for the mother, antigens encountered during pregnancy can elicit post-pregnancy sensitization. The classic example of this is the Rh^neg^ mother forming anti- Rh(D) antibodies to a Rh^+^ first child causing HDFN (hemolytic disease of the fetus and newborn) in the second. Another example is the anti- HLA antibodies formed in post-pregnancy against the baby's IPA that have long been a standard in the tissue-typing field.

For the child, the opposite appears to be true—in an exception to the strict rule of self/non-self discrimination, lifelong allo-tolerance to non-self antigens could result when they were encountered in the womb, and in milk during breast feeding, during the maturation of the immune system. The discovery of neonatal allo-tolerance was made possible by the observations of a young scientist named Ray Owen. Ray was following up previous studies by the lab head, Lillie, who had found in 1916 that in cows bearing male/female dizygotic twin calves, the female was often born as a “freemartin”, i.e. unable to produce milk due to the *in utero* exchange of blood with the male twin. This peculiar type of pregnancy led to the discovery of male androgens, and thus was of vital importance to the dairy industry [1]. Lilly's student Ray Owen found that not only did *in utero* exchange of blood between dizygotic cattle twins cause hormonal exchange, but that dizygotic twins with distinct inherited red blood cell types (analogous to the ABO system in man) were born with a stable 50:50 mixture of blood types (Owen [2]. The pioneer of transplantation biology, Peter Medawar, not knowing of Owen's discovery, found that skin grafts were exchanged between dizygotic cattle without rejection whereas 3rd party skin was rapidly rejected [3]. This discovery launched the field of transplantation, and enormous medical advances in treatment of end-stage organ failure followed soon after.

Non-inherited maternal antigens or NIMA, a leftover of maternal/fetal tolerance mechanisms in the newborn, can result in autologous immune suppression that profoundly impacts host allo-immunity in adult life [4] and promotes favorable organ transplant survival in NIMA-mismatched siblings [5,6]. Indeed exceptions to the maternal sensitization rule arise when the mother, herself the child of another woman, has thereby been exposed to certain “NIMA” as a fetus/newborn—these exceptions constitute the so-called “grandmother” effect in maternal-fetal tolerance [7].

## 2 What physical and immunological changes have evolved to respond to the need for a period of mutual maternal-fetal tolerance?

Due to mother's immune competence as an adult, and the developing immune system of the fetus within her, it was necessary for mechanisms of fetal-maternal immune tolerance to evolve in order for mammalian species to emerge. An excellent review of the evolution of localized maternal-fetal tolerance during development of the female womb is provided by Moffett and Loke [8]. I will later consider a second major evolutionary development, propagation of a stable truce between mother and fetus by a process that has come to be known in transplantation immunology as “infectious” tolerance Waldman [9].

As mammals began to emerge from reptilian lineages ∼120 million years ago, different species used 3 main types of approaches to reproduction. **Monotremes** (oviparous mammals; duck-billed platypus, etc.) had the eggs of the mother retained in the oviduct, but without a shell, after fertilization, until shortly before the egg is laid and the young hatches. However, monotreme mothers do feed milk to their offspring. In **Marsupials** (kangaroos, etc.) the mother produces eggs that are then fertilized and hatch within the oviduct at the 10-somite stage. Then the embryo implants briefly and superficially in the uterus, with a simple placenta, but no invasion of the uterine lining.

In **Eutherians**, the placenta is complex and shows varying degrees of invasiveness.

Three main subtypes of eutherian mammals are classified according to the extent of placental invasiveness. In epitheliochorial [for ex. bovine] mammals, fetal trophoblast cells attach to the surface epithelium of the uterus, but penetrate no farther. This feature, and the tendency of vascular anastomoses to form between dizgotic twin calves became the source of “experiments of nature” that gave rise to the discovery of male hormones in 1916 as well as the later discovery of neonatal immune tolerance in 1945[ summarized above]. In endotheliochorial mammals[for ex. African elephants, carnivores, etc.], trophoblast cells “invade” the uterine wall, but simply migrate to positions that “abut” maternal vessels, facilitating exchange of nutrients from maternal blood. Finally, in hemochorial mammals [mice, humans] the main feature is complex invasiveness. Here trophoblast cells emerge as a cell type distinct from the outer epithelium [chorion] of the amniote egg, and invade the uterine epithelium and endometrium extensively to establish a more intimate platform for maternal-fetal blood nutrient and waste product exchange. The hemochorial placenta is the most ancient of mammalian adaptations, arising first in the tiny(<100 mm in length) shrew that co-existed with dinosaurs. These pre-historic mammals managed to survive the Cretaceous-Tertiary (**K–T**) **extinction** event ∼ 66 million years ago that eliminated all of the dinosaur lineage on Earth except for the ancestors of modern-day birds.

In the hemachorial placenta a distinct lymphocyte population is recruited, the decidual NK cell [10].The task of mammalian pregnancy, particularly in the context of highly invasive placental cells, became one of a) immediate engagement of an inflammatory response at the initiation of pregnancy to ensure rapid blood supply at the site of embryo implantation; followed by b) construction of a physical separation/barrier between mother and the developing fetus; and c) expression of “decoy” MHC molecules[for ex., HLA- G,-E, and –C, expressed by the trophoblast cells, allowing a switch from a conventional T and B lymphocyte response to a specialized cell type (the decidual NK cell) responding to these MHC variants until pregnancy is firmly established. Later in pregnancy, as fetal and maternal stem cells “leak” through the placental barrier causing microchimerism, an immune regulation system in the mother and the fetus develops to suppress “classical” T and B cell allo-responses to these persisting cell migrants. The long-term goal of these features of mammalian pregnancy is the gradual development of an immune competence in the fetus. This immune competence, along with a selective immune regulation system developed in parallel, will allow him/her to avoid premature reactivity to maternal antigens without interfering with self-protection against pathogens after birth. Fulfillment of all the above requirements would allow a successful pregnancy for the mother, followed by return to normal immune self-defense. For the baby, development of nursing, locomotion, socialization & immunity in the neonate insures a successful start in life.

The evolutionary project of protecting mammalian pregnancy bears similarities to the more ancient project of separating the host from the gut bacteria necessary for digestion, while allowing full protection against systemic bacterial/viral infections. In both cases, the primary mechanisms were physical: for example the development of fetal encapsulation within the extra-embryonic membranes of the placenta, preventing maternal-fetal cellular interactions from occurring until very late in pregnancy [8] was similar to the strategy of development of a relatively impermeable barrier between beneficial gut microbes and host lymphocytes. In both cases a distinct type of immunity developed at the interface. In the case of the gut, a specialized B cell population that produces IgA was responsible for maintaining the “peace”, harmlessly escorting bacteria away from the blood and into the excrement. Similarly, the fetus is allowed to persist in the presence of a specialized decidual NK response, and a regulatory cell-controlled B and T cell response, without damage from maternal effector immunocytes until expulsion of baby and placenta at term ends the incursion by the semi-allogeneic transplant.

## 3 Role of IL35, TGFβ, ExATPase, and DC exosomes in fetal-maternal tolerance

Our lab has been interested in the local nature of transplantation tolerance, along with two related aspects, infectious tolerance and bi-directional regulation. The latter two features also play a critical role in the maternal-fetal tolerance arrangement, orchestrated by regulatory cell delivery of exosomal cytokines.

### 3.1 IL35

Soon after their discovery of IL35 in 2007 [11] Collison and Vignali noted that Ebi3 and (IL12) p35, the 2 subunits of IL35, were highly expressed in placental trophoblasts, suggesting that “IL-35 may be an immunomodulator at the feto-maternal border” [12], confirming an earlier report by Devergne on these subunits in human full-term normal placenta [13]. For the next 13 years, IL35 was thought of as a normal H_2_O-soluble cytokine, like IL10, involved in systemic immuno-suppression. However hints soon began to emerge that IL35 was quite different from other known cytokines. For example, its role in “infectious tolerance” was elucidated quite soon after its discovery— although initially produced by Foxp3+ Treg cells, it had the ability to convert conventional Foxp3^neg^ T cells into IL35 producers, termed “iTregs” [14]. In addition, the IL35 receptor required for this unusual “infectious” conversion event turned out to be different from the receptor required for its immunosuppressive action [15].

However, the secret of both the local and infectious nature of tolerance mediated via IL35 became clear with the work of Sullivan et al. [16]. In this paper, the authors found that IL35 was not H_2_O-soluble, like IL10, but rather was secreted as an exosomal product of Tregs and iTregs. The product was initially inactive as a [p35:Ebi3/2:1ratio] complex bound to CD81, a tetraspannin associated with exosomes, but could be converted to an active (1:1/p35:Ebi3) form after uptake by the target lymphocyte. Furthermore, bystander lymphocytes [for example, non-allo-specific T cells that happened to be in the vicinity], once exposed to IL35-bearing exosomes produced by specific Tregs and iTregs would take up these small extracellular vesicles, display IL35 on their surface, and themselves acquire the ability to suppress local immune responses. In other words, exosomal IL35 behaved as both a direct suppressor molecule, and as an agent of propagating local “infectious” tolerance [9].

### 3.2 TGFβ1

TGFβ1 is an extremely ancient cytokine [17] with various roles in pregnancy. It is secreted by the blastocyst, inducing apoptosis of uterine epithelial cells, suggesting that it plays an important role in embryonic signaling to the endometrium during implantation [18]. Like IL35, TGFβ1 has long been thought of as a H_2_O-soluble cytokine, released from Treg cells in its LAP-associated, “latent” form, but rapidly converted to a systemic immunosuppressive. Following up our work with IL35, we wished to determine if TGFβ1 might be yet another example of a cytokine derived from an exosome-bound precursor.

An important breakthrough in the biology of TGFβ1 was the discovery in 2009 of its anchoring to a membrane via association with glycoprotein A repetitions predominant (GARP), a transmembrane protein containing leucine rich repeats, which is present on the cell surface of Tregs [19]. The association of LAP-TGFβ1 with GARP, which has a *trans*-membrane region, meant not only that cell surface expression of LAP-TGFβ1 by Tregs was normal, answering a question originally raised by Warren Strober's lab [20]. It also suggested that a natural pathway for LAP/TGFβ1 secretion by Tregs might be as a complex with GARP on the surface of exosomes.

To test this hypothesis, we isolated exosomes from the serum of CBA-tolerized B6 mice, and from cultures of CBA re-stimulated lymphocytes, just as we had done in our studies of IL35. In these experiments, we tested for the secretion of LAP:TGFβ1 as a complex with GARP, in the exosome fraction. We found that, whereas all the induced IL10 was present in the 100,000×*g* supernatant, all of the TGFβ1:LAP-GARP complex was present in the 100,000×*g* precipitate, i.e. the exosome fraction [21].

### 3.3 Extracellular ATPase [CD39/CD73 complex]

As a control in our analysis of exosomal IL35, we found that Ebi3 knockout mice failed to generate IL35-bearing exosomes, while they continued to produce IL10 and TGFβ1 [16]. We used the CD39 inhibitor ARL67156. ARL67156 -sensitive inhibition was still present in exosomes of tolerized Ebi3 knockout mice, indicating an IL35-independent inhibitory pathway. Smyth et al. [22] had previously shown that besides the extracellular ATPase CD39, the extracellular ADPase CD73, also required for the generation of immunosuppressive adenosine, was present on exosomes released by Treg cells. Recent work has localized CD39/73 expression to cells of the human amnion, suggesting a direct role of purine metabolites as immunosuppressive agents protecting the human placenta/fetus from maternal T effector cells.

### 3.4 Allo-antigen presentation

In addition to inhibitory cytokines, exosomal products of maternal and fetal dendritic cells (DC) have a profound impact on fetal and maternal immunity. As discussed in Bracamonte-Baran et al. [7] exosomal products of DC include the basic ingredients for allo-antigen presentation. Whereas the so-called “indirect pathway” of allo-presentation, the key to late chronic rejection, relies on only cell-fragment or soluble alloantigen uptake by host DC, actual cellular microchimerism in the maternal or fetal host resulting from rare cell exchange during pregnancy opens up the possibility of non-classical, “semi-direct” allopresentation. This occurs when rare (microchimeric) cells produce enough exosomes to cause acquisition by host (maternal or fetal) DC and punctate allopresentation on the acquiring cell surface. Such “semi-direct” priming of the fetal host is coupled with a PD-L1-based inhibition of indirect allo-recognition and leads to the paradox of non-inherited maternal antigens (NIMA) in transplants between haplotype -mismatched siblings: more rapid acute rejection episodes, but less chronic rejection in long term follow-up [5]. In the mother, persistence of fetal microchimerism is expected to prime both indirect and semi-direct pathways leading to a higher rate of rejection. While this is generally true [23], if the inherited paternal antigen (IPA) of the fetus is the same as one that the pregnant women has herself previously been exposed to *in utero* due to a NIMA of grandmother, the outcome of sensitization may be avoided.

## 4 Summary

Putting these observations together, it would be safe to assume that more exosome-based adaptations for local immunosuppression, ideal for the fetal-maternal compromise, will be discovered in the future. In addition, the infectious nature of transplantation tolerance, a mystery since its original description by Waldman's lab in 1993 [9], may finally be solved by recent discoveries of a virus-sized, exosome-based, secretion by Tregs and other regulatory cells within the tolerant allograft. The fetus benefits from all of these natural exosome-based cytokines and alloantigen products in the form of a prolonged, stable semi-allograft both in their local distribution, and their “infective” qualities.

## 5 Conclusion

The evolution of the hemochorial placenta gave rise to the first small viviparous mammals. Since then (>125 million years ago) the course of maternal-fetal tolerance has been to develop novel strategies to protect the invasive placenta of the mammalian fetus. These strategies included physical barriers to limit maternal interaction with the developing fetus, as well as various immune suppressive mechanisms that have increased the survival of the semi-allogeneic fetus in the midst of a powerful mature maternal immune competence.

The advantages of the hemochorial placenta have been numerous: a) protection of the developing fetus during pregnancy, b) enabling a given mammalian species to tolerate a greater range of paternal interbreeding, and c) by physically isolating the fetus, greater opportunity for neonatal tolerance to develop. The answer to commensalism with gut-homing bacteria was similar, where a physical barrier along with a specialization of the immune system (IgA) allowed the benefits to digestion without an “alarmist” immune response.

One final note, although not dealt with in this review, a future research direction into bi-directional immunoregulation, a residue of maternal-fetal tolerance impacting HLA-identical, and a portion of HLA haploidentical kidney transplants [24], may hold important insights into the stability of the fetal “semi-allogeneic” transplant in mother.
